# A Modified Technique for Laparoscopic Spleen Preserving Distal Pancreatectomy

**DOI:** 10.1155/2018/1978362

**Published:** 2018-02-19

**Authors:** Andreas Papatriantafyllou, Theodoros Mavromatis, Theano Demestiha, Dimitrios Filippou, Panagiotis Skandalakis

**Affiliations:** ^1^3rd Department of Surgery, Evangelismos General Hospital, Athens, Greece; ^2^Department of Anatomy, Medical School, University of Athens, Athens, Greece

## Abstract

Spleen preserving laparoscopic distal pancreatectomy is considered as first choice operation for symptomatic benign or small malignant lesions located at the body or tail of the pancreas. The two main surgical techniques that have been proposed and widely adopted for spleen preserving laparoscopic distal pancreatectomy are the Warshaw and Kimura techniques. A novel modified approach for laparoscopic spleen preserving distal pancreatectomy is presented. The technique was initially performed in a 57-year-old female patient with mucinous cystadenoma. Following the surgical planes created by the fascia fusion and the organ rotation during embryogenesis (fascia of Toldt and renal fascia) with the patient in a right lateral decibutus position, the tumor was accessed retroperitoneally, without dividing the gastrocolic ligament and entering the lesser sac. The tail of the pancreas was mobilized anteriorly and medially, the lesion was visually identified and resected, and short gastric and left gastroepiploic vessels were preserved. We present the technical details and tips; we define the surgical anatomy of it and discuss the perioperative course of the patient as well as the possible benefits of the proposed technique. The proposed technique seems to be safe, easy to perform, and may present a promising alternative approach for patients with pancreatic disease that can be treated by laparoscopic pancreatectomy.

## 1. Introduction

Spleen preserving laparoscopic distal pancreatectomy is considered as first choice operation for patients with benign or small low-grade malignant tumors of the body or tail of the pancreas, such as cystic tumors, IPMN, or even chronic pancreatitis [1–4]. The advantages of spleen preserving laparoscopic distal pancreatectomy are those of shorter hospitalization and rehabilitation time, significantly less postoperative pain, less adhesions, and better aesthetic result, compared to open surgical procedures [3].

Most of the tumors that have been mentioned previously seem to be diagnosed more frequently nowadays due to the CT scan widespread use in the differential diagnosis of nonspecific abdominal pain (incidentalomas). In such cases, where the prognosis is excellent, it seems to be more beneficial for the patient to receive a minimal invasive spleen preserving operation so as to avoid lifetime immune deficiency and thrombocytosis. The potential advantages are obvious and include the decreased risk of infection or sepsis, while the necessity for chronic anticoagulant treatment (antiplatelet drugs) medication and chemoprophylaxis is minimized to rates of the general population [2, 5, 6].

The two surgical techniques that have been proposed and widely adopted for spleen preserving laparoscopic distal pancreatectomy are the Warshaw and the Kimura techniques. In Warshaw's technique, the spleen blood supply relies on the short gastric vessels and the left gastroepiploic artery which undertake the arterial flow instead of the ligated splenic vessels [7]. In Kimura technique, the splenic vessels are preserved during the operation to ensure the spleen blood supply [2]. In both techniques, the pancreas is accessed via the lesser sac after dividing the gastrocolic ligament. Many authors suggest the use of endoscopic ultrasound device or robotic surgical equipment to identify and locate the tumor [2, 3].

We present an alternative surgical approach for spleen preserving laparoscopic distal pancreatectomy which simplifies the preservation of the splenic blood supply, requires lesser mobilizations, and reduces the operating time. Furthermore, the high-cost equipment mentioned above (EUS and robotic equipment), which needs specialization and training to use, seems not to be mandatory.

## 2. Case Report

**Figure 1:**
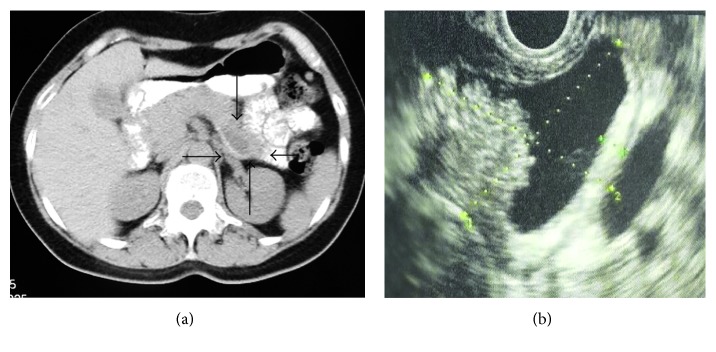
(a) CT scan of the patient in the case presented. The arrows point out the lesion in the tail of pancreas. (b) Endoscopic ultrasound (EUS) of the lesion followed by FNA biopsy.

A 57-year-old female patient, with no prior medical history, was admitted for cystic tumor of the tail of the pancreas. The patient complained for a nonspecific abdominal pain during the past month. Ultrasound exam revealed a cystic lesion of 3 cm diameter on the tail of the pancreas. These findings were confirmed by CT scan which showed a 3.7 cm cystic tumor on the body and tail of the pancreas (Figure 1(a)). Examination with endoscopic ultrasound (EUS) and FNA ensured our previous suspicion for mucinous cystic neoplasm (Figure 1(b)). From the blood analysis, the major findings were amylase 69 U/L, CEA 219.1 ng/mL, and Ca 19-9 115.8 U/mL.

The preoperative diagnosis was that of a mucinous neoplasm developed in the body and tail of the pancreas. The patient underwent laparoscopic distal pancreatectomy.

**Figure 2:**
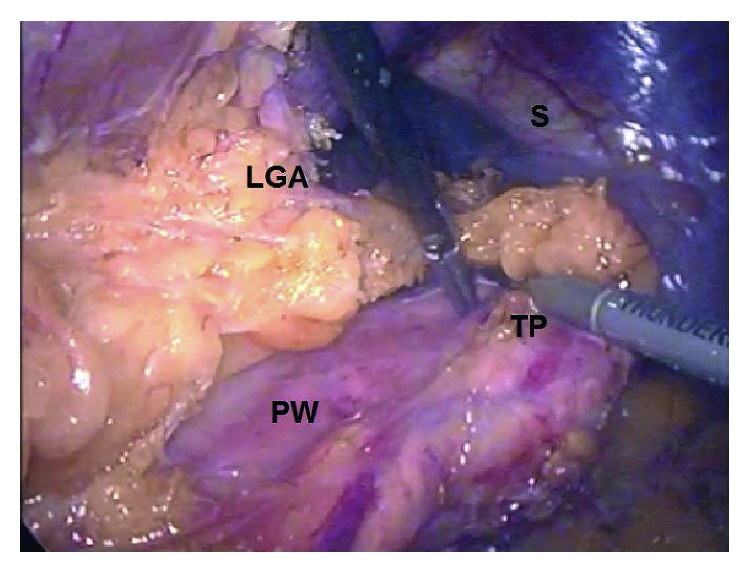
Intraoperative picture. S: spleen; TP: tail of the pancreas; LGA: left gastroepiploic artery; PW: the anterior surface of the pancreas which is also the posterior wall of the lesser sac.

**Figure 3:**
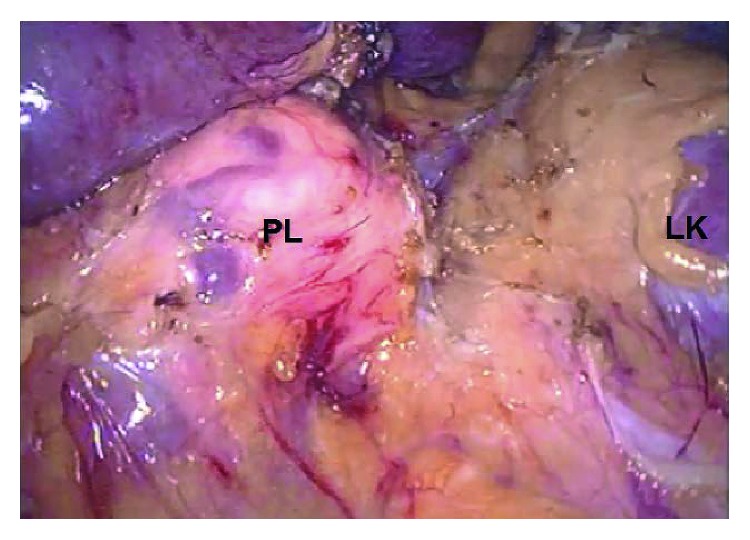
Intraoperative picture. LK: left kidney; S: medially mobilized spleen; PL: retroperitoneal lesion of the posterior pancreatic surface. The distal pancreas is medially mobilized.

The patient was placed in right lateral decubitus position and received general anesthesia with endotracheal intubation while a nasogastric tube was placed to evacuate the stomach. Initially, pneumoperitoneum was established using a Veress needle. Then three 11 mm trocars were inserted: the first on the midclavian line between the umbilicus and the anterior superior iliac spine; the second on the midclavian line 2 cm below the costal margin, and the third on the anterior axillary line, at the level of the umbilicus. The first trocar was used for the 30-degree laparoscopic camera and the second and the third for the laparoscopic instruments. A laparoscopic bipolar/ultrasonic device (Olympus Thunderbeat) used to coagulate, ligate, and divide tissue. After dividing the splenic ligaments, the spleen with the tail of the pancreas elevated anteriorly (Figure 2). This maneuver allows the identification of tumors that appear on the posterior surface of the pancreas due to the fact that the body and tail of the pancreas are only surrounded by the “fused fascia of Toldt” [2]. Therefore, the resection margin was defined by direct vision without the necessity of laparoscopic ultrasound (Figure 3). The pancreas then was resected by an endoscopic linear stapler medially to the lesion. An endocatch bag was used to remove the specimen from the abdomen. No bleeding, pancreatic fluid leak, or indications of splenic ischemia were noticed during the operation. A negative-low-pressure drain was placed, and the surgical wounds were closed as usual. The operating time was 210 minutes. This operating time can be compared to the mean operating time of other spleen preserving laparoscopic distal pancreatectomy techniques (200 minutes), although this was the first time this technique was performed. We assume that this time will be reduced when more experience will be gained.

**Figure 4:**
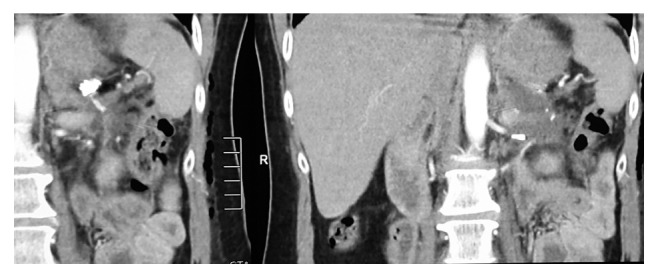
CT angiography performed on the tenth postoperative day showed that there was no splenic ischemia.

The patient did not present any minor or major complication during the postoperative period. The patient complained for nausea during the first and second postoperative days, so we decided to keep the nasogastric tube. On the third day, the nasogastric tube was removed, and on the fourth, day the patient received food and water per os. Blood serum amylase and amylase form the drain fluid were measured daily although there were not any abnormal results. The drain tube was removed on the seventh day, to safely exclude any pancreatic fluid leak and, therefore, formation of pancreatic fistula, generally appeared until postoperative day 5 to 6. A CT angiography that performed on the 10th postoperative day to evaluate the spleen blood supply did not indicate any signs of splenic ischemia or infraction [7] (Figure 4).

**Figure 5:**
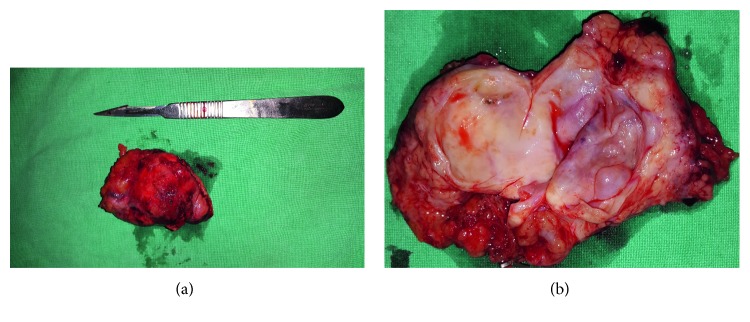
Specimen (a) after extraction and (b) after being divided. The dual cystic lesion is visible.

The patient was discharged on the 11th postoperative day. The patient lives in a very promote mountainous village with lack of health services and difficulties in moving, so we decided to remain hospitalized until the required imaging exams complete. That is the reason for the longer hospitalization time. The histological analysis of the specimen suggested mucinous cystadenoma with low-grade dysplasia. The specimen's dimensions were 4.5 × 4 × 2.5 cm. Most of the specimen's tissue was a dual-cystic lesion (max. *d*∼4.5 cm), regionally occupied by ovarian—like ovarian stromal tissue, a-inhibin—PR positive. The epithelium immunophenotype was EMA(+), CK18-8(+), CA19-9(+), MUC-1(+), MUC-6(+), and MUC-5AC(±) (Figure 5). Five lymph nodes which were also extracted showed only mild inflammatory lesions. The resection margins were free of disease. No signs of splenic ischemia or infraction were presented on a CT angiography that was performed one year postoperatively, and the platelet counts on the blood tests were within normal range.

## 3. Discussion

**Figure 6:**
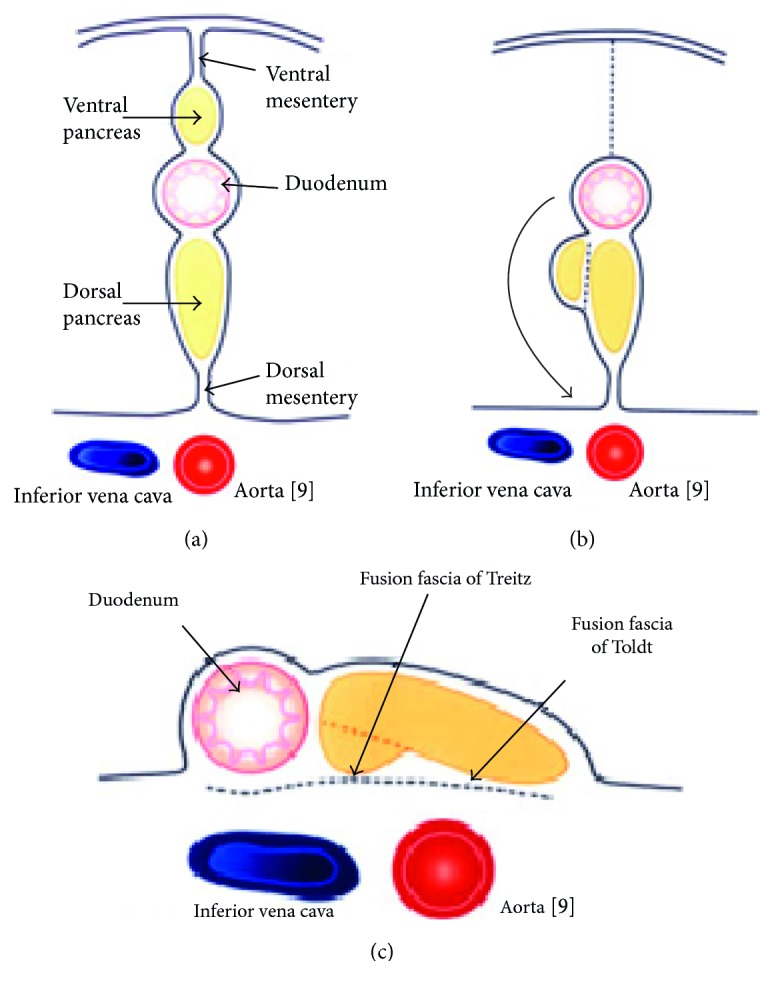
Embryological formation of the surgical planes allowing the distal pancreatectomy as described. Rotating around the duodenum ventral pancreas and the dorsal pancreas form the pancreas. The rotation of the pancreas and the duodenum cause the fusion of fascias of Toldt and Treitz [9].

During embryological development, pancreas becomes a retroperitoneal organ secondarily. It is initially divided into two buds, representing the ventral and dorsal anlagen. The ventral anlagen of the pancreas then moves around the duodenum until it comes into contact with the dorsal bud. The membrane of the ventral bud and the one of the inferior vena cava and abdominal aorta become fused, and this is reflected by the term “fusion fascia.” The fusion fascia of the head of the pancreas is known as the “fusion fascia of Treitz,” and the fusion fascia of the body and tail of the pancreas is called the “fusion fascia of Toldt.” The “fusion fascia of Toldt,” lying above the renal fascia, is completely surrounding the body and tail of the pancreas. The fused fascias are the anatomic surgical planes allowing the mobility of the pancreas from the retroperitoneal area through a retroperitoneal (or anterior interfascial) plane [2, 8] (Figure 6).

The splenic vessels are running along the superior surface of the pancreas and into the hilum of the spleen. Other anatomical structures that the surgeon should be familiar with are the ligaments between the pancreas, the colon, the left kidney, and the diaphragm. These ligaments have to be divided for better exposure and control of the splenic vessels to obtain retroperitoneal access to the pancreas. The fact that the pancreas tail is not attached to the retroperitoneum, but actually it is rather mobile helps our technique. Following the presented spleen preserving laparoscopic distal pancreatectomy technique, there is not necessary to enter the lesser sac by dividing the gastrocolic ligament, in order to mobilize the pancreas.

After establishing pneumoperitoneum, three 11 mm ports are inserted as mentioned above. The splenic flexure is mobilized and depressed by dividing the lateral peritoneum across the “line of Toldt” towards the descending and transverse colon. The splenorenal, splenophrenic and pancreaticosplenic ligaments are divided so that complete mobilization of the spleen is accomplished. When this maneuver is performed with the patient in the particular position (gravity—weight of the spleen and the tail of the pancreas), this allows the appropriate traction for further mobilization of the pancreas up to its body in the retroperitoneal space anteriorly, across the renal fascia (Gerota's), and the fused fascia of Toldt. The transverse mesocolon is mobilized away from the pancreatic inferior-anterior surface. Lesser sac is now accessible posteriorly (Figure 2). On the superior surface of the pancreas, after dividing the fused fascia of Toldt, it is possible to mobilize the pancreas and the splenic vessels from the retroperitoneal structures by carefully ligating and dividing the splenic vessels medially from the resection margin and laterally between the pancreatic tail and the spleen using titanium clips. The gastrocolic and gastrosplenic ligaments as well as short gastric vessels and the left gastroepiploic artery are preserved, and the blood supply to the spleen remains sufficient. Even small tumors on the posterior surface of the pancreas are identified under direct vision by simply elevating the fully mobilized pancreatic tail and body (Figure 3). The pancreas can now be resected by an endoscopic linear stapler medially to the lesion and removed by an endocatch bag from the abdomen to avoid spoiling.

The proposed alternative surgical approach for spleen preserving laparoscopic distal pancreatectomy consists on exclusively retroperitoneal mobilization of the pancreas, without dividing the gastrocolic ligament to gain access to the lesser sac. When this maneuver is performed with the patient in the particular position (gravity—weight of the spleen and the tail of the pancreas) allows the appropriate traction for further mobilization of the pancreas up to its body in the retroperitoneal space anteriorly, across the renal fascia (Gerota's), and the fused fascia of Toldt [2, 4, 5]. Therefore, the resection margin can be defined by direct vision without the necessity of laparoscopic ultrasound. The use of such devices is essential in other techniques that access the pancreas anteriorly through the lesser sac because of the existence of the peritoneum that covers the anterior surface of the pancreas [10–12].

The preservation of the short gastric vessels and the left gastroepiploic artery is reassured because the gastrocolic and gastrosplenic ligaments that contain those vessels are left untouched.

Any possible bleeding from minor arterial branches of the splenic artery at the level of the splenic hilum is easily manageable because the, already mobile, pancreatic tail is further more mobilized away from the splenic hilum [12]. According to all references and recent data, robotic spleen preserving distal pancreatectomy techniques sacrifice more healthy pancreatic tissue than necessary. This fact does not come along with the overall idea of minimal invasive excision of benign or low grade malignancy lesions [10]. As far as it concerns obese patients, laparoscopic distal pancreatectomy does not increase the possibility of pancreatic fistula [2, 3, 10]. Furthermore, the right side down lying operative position offers better exposure of the surgical field and facilitates mobilizations and maneuvers.

The proposed technique presents some significant advantages. Using a medial to lateral technique (Kimura's technique) for pancreas and splenic vessels, mobilization is more demanding, and laparoscopic intraoperative ultrasound may be required to recognize the lesion and identify the margins. The use of such a device increases the cost of the procedure and requires special skills and training for its use. The operating time is longer and blood loss is higher compared to a lateral to medial approach [11–14]. In the past six years, several authors have also proposed a lateral to medial approach for laparoscopic spleen preserving distal pancreatectomy, using different techniques to mobilize and preserve the splenic vessels, especially the splenic vein which is usually embedded in the pancreatic parenchyma except the tail of the pancreas.

In the “straightened splenic vessel” technique, the vessels are identified in a lateral to medial approach, but they are dissected from the pancreas in the opposite direction [12]. In most references, five ports and a flexible laparoscope are used most commonly instead of three ports and a 30-degree laparoscope, and the patient is placed in a supine position. The traction applied to the splenic vessels can cause injury or thrombosis leading to splenectomy.

Nakamura et al. in 2011 have used a technique similar to the one we proposed. In their report, the lesser sac was accessed without dividing the gastrocolic ligament and all mobilizations were accomplished “lateral to medial” [13]. The difference consists on the use of laparoscopic ultrasound and five ports that they used to perform the procedure, and the splenic artery and vein were also preserved. In the same article, they also describe a laparoscopic Warshaw method (the splenic artery and vein were divided), similar to the technique we propose, but the gastrocolic ligament was divided, and a laparoscopic ultrasound device and five ports were used. The patient was placed in a supine position in both techniques, instead of the right lateral decubitus position that we used in our technique.

Strickland et al. in 2014 proposed a technique with significant similarities to ours, but he used five trocars to complete the operation like Nakamura instead of three that we used [14].

## 4. Conclusions

The proposed technical modification can be performed even in cases of prior abdominal operation, where adhesions exist, due to the retroperitoneal approach. This alternative technique is actually a simplified version and modification of already existing procedures, but present significant advantages. It does not require high-cost equipment or devices that need specific training. The intraoperative time, the blood loss, and the total complication rate may be significantly decreased. Unnecessary resection of normal pancreatic parenchyma is limited, and the risk of pancreatic insufficiency is minimized. Finally, short gastric vessels and the left gastroepiploic artery are preserved, keeping spleen's blood supply, avoiding the postsplenectomy immunological and hematological complications.

The initial experience suggests that the described technique is safe, easy to learn and perform and may represent an efficient alternative in most, if not all, distal pancreatic lesion cases. Its possible advantages suggest that this technical modification may be considered as a useful and reliable alternative approach for patients with benign or low malignancy tumors of the tail of the pancreas that have indication for laparoscopic spleen preserving distal pancreatectomy.

## Data Availability

Data are available on request from Andreas Papatriantafyllou (andpapat@hotmail.com).
